# Shared Knowledge and Verbal Communication in Football: Changes in Team Cognition Through Collective Training

**DOI:** 10.3389/fpsyg.2019.00077

**Published:** 2019-01-31

**Authors:** Marc A. Blaser, Roland Seiler

**Affiliations:** Institute of Sport Science, University of Bern, Bern, Switzerland

**Keywords:** team cognition, shared knowledge, verbal communication, collective training, team coordination, football

## Abstract

One of the psychological mechanisms that contribute to effective and efficient team actions is team cognition, defined either as shared knowledge states about game situations, teammates’ skills, and action probabilities or direct communication processes in the team action itself. Particularly in interactive team sports (e.g., football), characterized by highly complex, dynamic, and uncertain situations, sharing a common understanding concerning potential future actions and how to coordinate these actions may be an advantage. Otherwise, team members must communicate their thoughts and ideas on the fly, which might be impossible due to time pressure, cognitive costs or noisy environments. This study examined if shared knowledge and verbal communication change through collective training. Forty-six under-18 and under-21 youth football players performed a football task in teams of two. The task consisted of passing and running elements common in football. After a training phase, and before two testing phases, players evaluated their actions and the actions of their assigned teammate regarding action type, location, and timing. Out of these evaluations, two indices of common understanding were computed. Furthermore, verbal communication during the task was video-and audio-recorded. Data analysis showed that shared knowledge considerably increased over time and with practice. Simultaneously, overall verbal communication and verbal communication consisting of orienting information was significantly reduced. Additionally, there was a tendency for a correlation that when shared knowledge increased, orienting verbal communication decreased. Overall, the players used orienting communications the most (77%). The study revealed that shared knowledge states and verbal communication change through collective training and that there might be a relation between the level of shared knowledge and the use of orienting verbal communication. Further studies in and off the field are needed to disentangle the complex interplay of team cognitions.

## Introduction

Whether playing a solid, insuperable defense; executing a fluid counterattack; or finishing a play with a blind no-look pass to the freestanding teammate to score, one of the psychological mechanisms contributing to effective performance in team sports like football is team cognition (Gray et al., 2017). Team cognition highlights the social-cognitive activity that emerges before, during, and after interactions between team members (Eccles and Tenenbaum, 2004; McNeese et al., 2016). Team cognition can be viewed either as shared knowledge or as ongoing team communication (McNeese et al., 2016). The first perspective is static and pertains to “knowledge structure(s) held by each member of a team that enables them to form accurate explanations and expectations for the team and task, and in turn, to coordinate their actions and adapt their behavior to demands of the task and other team members” (Cannon-Bowers et al., 1993, p. 228). The latter perspective is rather dynamic. Moreover, communication among team members is considered cognitive processing at the team level. When it comes to effective team performance, both levels of cognition come into play, “meaning that individual cognition is required and necessary for team level cognition to derive” (McNeese et al., 2015, p. 1217) and both levels, shared knowledge and communication processes, are two crucial elements to coordinate team actions.

While shared knowledge issues were studied a lot in the military and I/O context (see for example Mathieu et al., 2000; Langan-Fox et al., 2001), there is a lack of empirical studies on team cognition in sports (Cannon-Bowers and Bowers, 2006) and, more specifically in football (Gershgoren et al., 2013). When team cognition has been studied, it was mainly from the perspective of shared knowledge (see Blickensderfer et al., 2010; Bourbousson et al., 2011). Few studies have been conducted on communication issues in sports teams (see Hanin, 1992; Lausic et al., 2009; LeCouteur and Feo, 2011). To our knowledge, so far no study integrating both perspectives, that is shared knowledge and communication exists. In general, empirical research on team cognition and team coordination in sports is still in its infancy (Eccles and Tran-Turner, 2014). Therefore, this study seeks to empirically test theoretical claims trying to implement both perspectives in the context of football. This seems to be a promising new way of studying the topic (see Gorman, 2014; McNeese et al., 2016).

*Being in sync* or *being on the same page* was claimed to have a significant role in the performance of team sports (McNeese et al., 2016). In interactive team sports, such as football, players perform in a highly dynamic environment involving time constraints and physiological restrictions (Gershgoren et al., 2013). Therefore, coordination breakdowns and failures (e.g., a bad pass) regularly occur. However, particularly in high-pressure situations with little time for overt communication, team cognition in the form of shared knowledge becomes crucial (Rentsch and Davenport, 2006). This knowledge allows players to coordinate their actions implicitly, without investing cognitive effort in verbalizing their thoughts, plans, and expectations.

Rentsch and Davenport (2006) identify several forms of shared knowledge, two of which are central to this study: the meta-accuracy and reciprocity form. Meta-accuracy is the degree to which a team member’s understanding of how another teammate views him- or herself with regard to a skill or specific action is accurate. Reciprocity represents how similar team members are in their views of each other, regarding complementary actions (e.g., playing a pass and receiving a pass). Thus, a player can have accurate expectations about the actions of his or her teammate, when they reflect their own thoughts and when team members have reciprocal expectations. For example, a midfielder is better able to adapt the timing of his pass when he knows the striker’s preferences for receiving the pass. This interaction is even more likely to be well coordinated when the mutual expectations about each other’s actions are accurate or true, meaning that the expectations fit the players’ intentions.

A key function of team cognition is the coordination of players’ individual actions “so that, when they are combined, they are in suitable relation for the most effective result” (Eccles and Tran, 2012, p. 32). Relation means that team members’ actions must be arranged correctly according to three dimensions: action type (e.g., what to do or how to do an action), timing (e.g., when to do an action), and location (e.g., where to do an action) (Eccles, 2010). It is assumed, that when team members do have similar knowledge about these dimensions, the coordination of the single actions is effective and permits a flowing and effortless team action. For example, the striker’s knowledge of how, when, and where the midfielder is going to act must be like the midfielder’s knowledge of how, when and where the striker is going to act and vice versa. If the mismatch in the mutual expectations is too significant, coordination breakdowns (e.g., turnover) may occur due to processes of false anticipation and adaptation (see also Steiner, 1972).

Theoretically, there are two central paths of establishing shared knowledge: (i) rather time-consuming procedures before a game (e.g., tactics) via explicit planning and other training forms like cross-training, training for adaptability or self-correction training (see Seiler, 2014) and (ii) routinisation during actual play or collective training (Eccles and Tran, 2012). Explicit planning involves coaches or other team leaders communicating plans, plays, and tactics at various levels of action (e.g., outcomes, designs, procedures, and operations) off the field or during breaks. In contrast, shared knowledge established through routinisation develops as a result of experiences team members make during dyadic interactions (Kozlowski et al., 1999; Pearsall et al., 2010). In essence, it is knowledge about situational probabilities; “that is, knowledge of what the team and its individual members are likely to do in … a given game situation” (Eccles and Tran, 2012, p. 33). So far, no empirical study has investigated the effects of routinisation or training on shared knowledge in sport.

In less pressurized game situations, where cognitive effort is low, verbal communication is an explicit means to create an effective sequence of single actions (Eccles and Tenenbaum, 2004). “Effective communication is critical to the success of any team or organization and its members” (Yukelson, 2015, S. 140). Along with non-verbal communication and behavior (e.g., body language; Seiler et al., 2018), verbal communication seems to be crucial in team sports (Sullivan et al., 2014). Furthermore, *in game communication* tends to be more relevant than *out of game communication*, because it is assumed more and directly related to performance outcomes (Sullivan et al., 2014; Tenenbaum and Gershgoren, 2014).

Hanin (1992) described four types of *in game communication*- orientation, stimulation, evaluation, and task-irrelevant. Orientation refers to those communications by teammates regarding planning and coordinating interactions (e.g., what do to, how, when, and where). Stimulating is defined as messages motivating partners to maintain or increase/decrease activity levels with no reference to what to do and how to do it. Evaluation means positive or negative statements about owns or players’ actions or behavior. Task-irrelevant communications are positive or negative messages with no bearing on performance. The few studies about in game communication in team sports (Hanin, 1992; Lausic et al., 2009; LeCouteur and Feo, 2011) have shown that there exist specific patterns around the flow of play, that the content is usually task-focused. Additionally, messages have typically a length of one or two words and may be repetitive.

This study focused on shared knowledge based on the three coordination parameters action type, location, and timing required to create a well-coordinated team action. We aimed at the acquisition through collective training, which accounts for a dynamic, situated form of establishing shared knowledge in football. More specifically, this research focused on changes of meta-accuracy and reciprocity knowledge as well as verbal communication behavior through collective training. We hypothesized that shared knowledge becomes more similar over time and with training and that, consequently, the use of verbal communication, especially containing orienting information, decreases due to a better common understanding about coordinating actions that has no longer to be communicated. Therefore, a small and negative correlation between each of the two knowledge forms and the use of orienting verbal communication was expected (see also Eccles and Tenenbaum, 2004).

## Materials and Methods

### Participants

Forty-six male elite junior football players (*M*_age_ = 17.04, *SD* = 1.13) playing for the under-18 and under-21 teams of a youth football academy of a club in the highest league in Switzerland participated in the study. The mean number of years of competitive playing and club training experience was 11.38 (*SD* = 1.67). Players have been in the same team at least since the beginning of the season. This means that they have known each other from the normal training and competitions for at least 5 months (*M* = 15.00; *SD* = 11.10). For our study, dyads were formed out of the participants. These dyads were formed within their respective age groups, with the 46 participants resulting in 23 dyads (16 dyads of under-18 players and 7 dyads of under-21 players).

This study was carried out in accordance with the recommendations of the University of Bern Ethical Committee with written informed consent from all subjects. All subjects and their parents gave written informed consent in accordance with the Declaration of Helsinki. The University of Bern’s Ethical Committee approved the protocol.

### Task and Procedure

**FIGURE 1:**
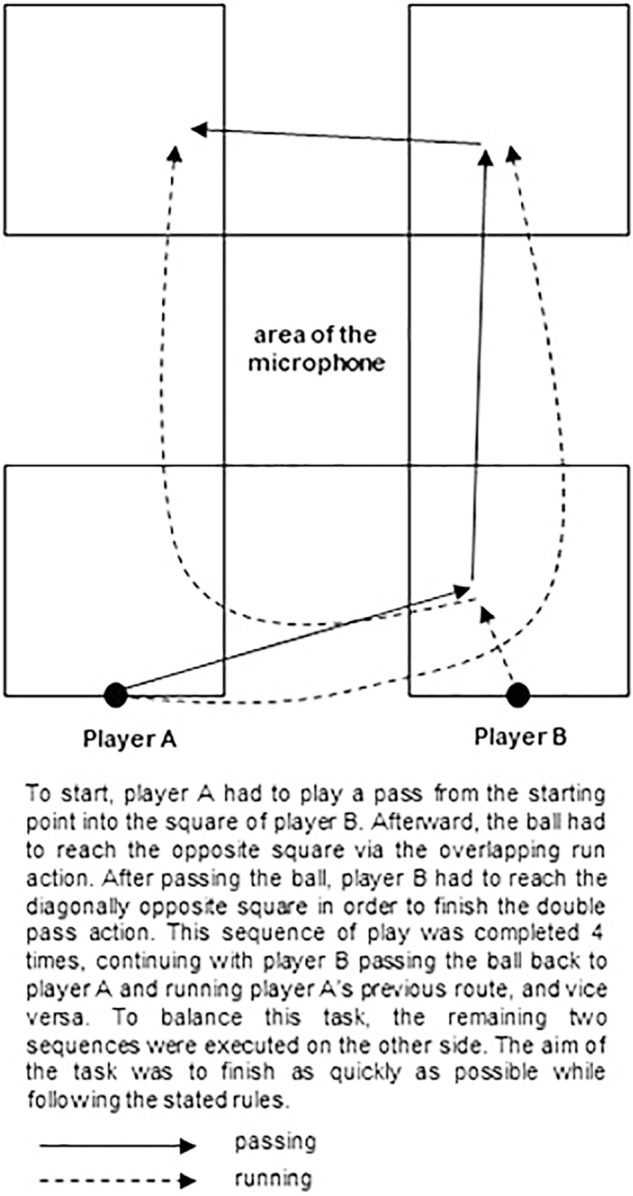
Illustration of the team task.

This study utilizes a repeated measurements design with two measuring points separated by approximately 15 min. Due to the complexity of football games and the dynamic change of situations, as well as the restricted possibilities for measuring shared knowledge and communication during a real game, we designed a task for two players including the crucial, tactical, and technical elements of an *overlapping run* and a *double pass* (see Figure 1 for a detailed description).

The procedure before and throughout the measuring points was the following: After a welcoming, players were introduced to the standardized task and had the opportunity to practice the elements. Subsequently, they performed two competitive practice runs in teams of two. Based on their experiences in these runs, each player evaluated his actions by action type (e.g., passing velocity; *how* to play), location (e.g., *where* to play), and timing (e.g., *when* to play) via situation-specific questions (see also Section instruments and measures). Afterwards, players were informed about their new teammates for the real experiment. Thus, every player had to evaluate his newly assigned teammate based on the same questions, that is, at what speed, where and when he expects the teammate to play and receive the ball. These ratings were based on previous training and match experiences. Teams were randomly assigned in that way, that a “more skilled” player (rating A) was playing with a “less skilled” player (rating B). The responsible team managers/coaches made these player ratings beforehand. Then, these dyads completed three times a competitive run to finish the first measuring point. Upon termination, they independently answered the same questions for the second time (this lasted approximately 15 min in average) and completed another three competitive runs in the same teams to finish the second measuring point.

The players were only allowed to speak together during the task. To prevent the creation of shared knowledge in-between the runs and the measuring points, players were separated to disable verbal communication and planning. Therefore, we could assume that shared knowledge was acquired through collectively training together.

### Instruments and Measures

So far, scales developed with the aim of measuring shared knowledge about teams (for example, Johnson et al., 2007) include general team and task knowledge, attitudes, and communication skills. Based on Cooke et al.’s (2013) postulation that “team cognition is inextricably tied to context” (p. 273), we, together with football experts, created ten situation-specific single items to gather relevant shared knowledge related to the specific football team task. Each player rated his actions and the actions of his teammate by the three coordination parameters action type (1 item overall), location (two items, one item for each situation; *overlapping run* and *double pass*) and timing (two items, one item for each situation) from the pass giver’s perspective (e.g., “*how* fast do you/does he play passes in the task?”; “*where* do you/does he play the pass in the *overlapping run*/*double pass* situation?”; “*when* do you/does he play the pass in the *overlapping run/double pass* situation?”) and from the pass receiver’s perspective (e.g., “*how* good is your/is his skill to receive passes in the task?”; “*where* do you/does he demand/desire the pass in the *overlapping/double pass* situation?”; “*when* do you/does he demand/desire the pass in the *overlapping/double pass* situation?”). In total, thus, ten self-evaluative and ten foreign-evaluative items resulted. These questions covering the two main team actions were answered on a corresponding 6-point Likert scale (e.g., *how*: “extremely fast” to “not fast at all”/“extremely good” to “not good at all”; *where*: “extremely precise into the foot of my teammate” to “extremely steep into the run of my teammate”/“extremely precise into my/his foot” to “extremely steep into my/his run”; *when*: “as soon as I/he received the ball and my/his teammate is still in the same square from where he passed me the ball” to “as soon as my teammate/he stays in the square we have to play in”/“as soon as I/he passed the ball and still stay(s) in the same square” to “as soon as I/he stay(s) in the square we have to play in”). To facilitate better understanding, illustrations accompanied the questions about action location and action timing (see Appendix for an example).

A 360-degree microphone placed in the middle of the task construction measured verbal communications. The microphone was linked with a standard video camera.

### Data Analysis

We used the answers of the situation-specific questions to compute a meta-accuracy and a reciprocity accordance index. For the former index, self-evaluation ratings of each item from player A were calculated together with player B’s external evaluations about the same perspective (e.g., pass giver) of player A and vice versa. For example the self-evaluation of player A is: “I play very fast passes” [Likert-Score (LS) = 5] and player B’s evaluation of player A is: “He plays rather fast passes” (LS = 4). In turn the self-evaluation of player B is: “I play rather less fast passes” (LS = 3) and player A’s evaluation of player B is: “He plays not fast passes” (LS = 2). The sum of the absolute differences between these 2 × 2 values over all ten items constructed the team meta-accuracy index [e.g., |(5-4)| + |(3-2)| = |2|]. For the reciprocity index, self-evaluation ratings of each item were calculated together with the same player’s evaluations about his teammate concerning the complementary perspectives and actions and vice versa. For example the self-evaluation of player A as the pass giver is: “In the *overlapping run* situation, I play the pass very precise into the foot of my teammate” (LS = 5) and the evaluation of player A about player B as a pass receiver is: “In the *overlapping run* situation, he demands/desires the pass very precise into the foot” (LS = 5). In turn the self-evaluation of player B as the pass giver is: “In the *overlapping run* situation, I play the pass very steep into the run of my teammate” (LS = 2) and the evaluation of player B about player A as a pass receiver is: “In the *overlapping run* situation, he demands/desires the pass very precise into the foot” (LS = 5). Accordingly, the sum of the absolute differences between these 2 × 2 values over all ten items resulted in the team reciprocity index [e.g., |(5-5)| + |(2-5)| = |3|]. In general, when generating these indices, the lower the absolute differences over all items, the more similar the mutual understanding. To analyze the changes of verbal communication, two independent persons counted the frequency of meaningful communication units per measuring point (Intraclass correlation; Cronbach’s α = 0.985, *p* < 0.001). A meaningful unit was defined as a self-consisted and closed message (e.g., “Yes, play!” or “go, go, go!”; see Table 1 for more examples). Furthermore, based on the classification and definitions by Hanin (1992), one of the raters categorized these meaningful communication units into Hanin’s schema. Ambiguous messages were separately discussed with the first author and classified appropriately. In the course of the classification process, Hanin’s (1992) evaluation category was split in positive evaluation and negative evaluation and extended by a mixed and unclear (not apprehended messages) category. All the variables were then analyzed using ANOVA repeated measures with time as a within subject factor with two levels. For correlational analysis, we used Pearson coefficients. For the correlational analysis regarding the relation between the indices and the use of orienting verbal communication, *post hoc* power analysis was quite low (1-β = 0.26). Therefore, the chances to accept the alternative hypothesis were small. For all other analyses, power was good (1-β ≥ 0.80).

**Table 1:** Sample expressions and mean percentage of use for verbal communication dimensions (total expressions/communication units *N* = 710).

Dimension	Examples		
Orientation (77.0%)	“Yes + *Name*!”	“Yes, play!”	“Run deep!”
Stimulation (6.0%)	“Go, go, go!”	“Come on!”	“Fast!”
Positive evaluation (1.9%)	“Perfect!”	“Well played!”	“Nice pass!”
Negative evaluation (5.4%)	“Fu^∗∗^!”	“Damn!”	“No!”
Task-irrelevant (4.6%)	“Oahhh!”	“Dude!”	“Ahhh!”
Mixed (2.6%)	“Yes go!”	“Nice, go!”	“Yes, awesome!”
Unclear (2.5%)			


## Results

Table 2 shows the mean accordance index scores of both indices, as well as the mean of used verbal communication units split up by category at t1 and t2. ANOVA revealed a small significant main effect of time for the meta-accuracy [*F*(1,22) = 4.43, *p* = 0.047, $\eta_{p}^{2}$ = 0.17] and a strong significant effect of time for the reciprocity index [*F*(1,22) = 34.9, *p* < 0.001, $\eta_{p}^{2}$ = 0.61], meaning that the dyads became more similar over time in their evaluations. For the overall verbal communication data, ANOVA revealed a significant main effect of time [*F*(1,22) = 9.71, *p* = 0.005, $\eta_{p}^{2}$ = 0.31], where verbal communication decreased from t1 to t2. Category wise, orienting verbal communications became fewer [*F*(1,22) = 21.2, *p* < 0.001, $\eta_{p}^{2}$ = 0.49], whereas all the other categories (stimulating, evaluative positive, evaluative negative, task irrelevant, mixed, and unclear) did not significantly change over time and training together. Either way, the orienting category was by far the most used one in the team task, accounting for 82% of all units at t1 and 72% at t2, respectively (see Tables 1, 2).

**Table 2:** Accordance index scores and number of communication units at the two measurement points (*N* = 23 pairs; means and standard deviations).

Measure	t1		t2	
	*M*	*SD*	*M*	*SD*
Meta-accuracy index score	20.61	5.91	18.52*	5.95
Reciprocity index score	14.61	3.82	10.57*	3.40
Overall verbal				
Communication use (in units)	5.65	3.52	4.62*	3.43
Orientation	4.58	2.95	3.34*	2.45
Stimulation	0.30	0.27	0.36	0.49
Positive evaluation	0.04	0.11	0.14	0.30
Negative evaluation	0.20	0.30	0.29	0.35
Task-irrelevant	0.22	0.33	0.24	0.30
Mixed	0.10	0.18	0.13	0.26
Unclear	0.09	0.18	0.14	0.22


*^∗^sig, difference between measuring points, p < 0.05.*

Correlation analysis showed a positive-moderate significant correlation between the differences of both accordance indices (*r* = 0.35, *p* < 0.05). Correlational analysis of the differences between t1 and t2 in the variables *accordance indices* and *overall verbal communication* revealed no significant correlations. However, a tendentious and weak relation between the indices and orienting verbal communication use could be found (for meta-accuracy *r* = 0.22, *p* = 0.15 and for reciprocity *r* = 0.22, *p* = 0.15), in the sense that the more similar the indices became, the less orienting communication was used (see Table 3). For all analyses, team age (e.g., how long team members knew each other) did not affect the results.

**Table 3:** Pearson correlations of the differences between the measurement points of the dependent variables (*N* = 23 pairs).

Measure	1	2	3
(1) Difference of meta-accuracy index	1		
(2) Difference of reciprocity index	0.35*	1	
(3) Difference of orienting verbal communication units	0.22	0.22	1


*^∗^p < 0.05.*

## Discussion

The study addressed the changing of shared knowledge and verbal communication through collective training. To our knowledge, this was the first attempt that investigated team cognition in team sport under an integrative perspective in the field, and the first attempt to assess the similarity of different forms of shared knowledge as proposed by Rentsch and Davenport (2006). Our data showed that shared knowledge aligned after performing together. In fact, both indices we used to assess shared knowledge, that is meta-accuracy and reciprocity, were adopted after carrying out the task together in comparison to the sole evaluations made beforehand. Although the players had known each other for at least 5 months, their evaluations became more accurate after practicing the specific task together for only a few times. This indicates that situation-specific shared understandings emerge with effective, situation-specific collective training. A general understanding that may exist because of familiarity reasons (knowing each other beforehand from training and match experience) could therefore be underpinned by a more detailed and specific knowledge. Given that football is a very open and dynamic team sport, training for a common understanding of all possible situations is difficult, if not impossible. None-the-less, this method of training and acquisition of shared knowledge may prove beneficial in standard and recurrent situations (e.g., corners and majority situations), where team actions like *double passing* and *overlapping run* are often used strategies to overcome the opponent’s defense. At the same time, counter actions of the opponent may interrupt every team action. In this case, an optimal interplay between a general and a situation-specific shared knowledge may be the most beneficial, where situational probabilities could be balanced. Additionally, given how quickly shared knowledge changes through collective training on a specific task, this method may be more efficient than time-consuming methods like cross-training, training for adaptability, and team self-correction training (see Seiler, 2014). Therefore, a practical advice for coaches and managers could be to consciously practice certain offensive and defensive game scenarios in order to establish a sense of blind understanding.

The mean use of verbal communication between the players decreased over time. Though this aligns with theoretical claims (Entin and Serfaty, 1999; Eccles and Tenenbaum, 2004), the assumed correlation between increase of shared knowledge and the decrease in verbal communication was only implied. This finding indicates two independent team cognition mechanisms that can be used for efficient team coordination, given enough available cognitive capacity (e.g., game situations with little time pressure and without interfering opponents). The use of verbal communication could, therefore, underpin shared knowledge. At the same time, we could strengthen that task-specific, orienting information during team action in interactive team sports like football is the most relevant. This is in line with Hanin’s (1992) observations in handball and basketball. Furthermore, our results corroborate the findings of LeCouteur and Feo (2011) that verbal expressions during team sport actions are in the majority of cases short and may have repetitive character.

Another tendentious, but not expected result of the study was, that the fewer orienting information is used the more other kinds of information are sent. Thus, we could assume that once a certain state of common understanding and shared knowledge is achieved, there might be capacity for other relevant issues like managing activity level. Giving more evaluative feedback then could also be used to refine shared knowledge states (Bourbousson et al., 2011). This assumption would weaken the claim that shared knowledge is only a static concept. Due to dynamic *in game communication*, shared knowledge states could adapt. However, these claims have to be empirically tested and theoretically integrated in current or new models.

Our study was not designed in the style of a typical learning paradigm. More specifically, we did not intend to test for retention effects in the communication pattern acquired, nor did we use a completely novel task with unknown partners in the experiment. In contrast, our study aimed at investigating the changes in both communication and shared knowledge about the partner in a real field situation in dyads. The results confirm that even through short periods of joint action a routinisation process may be initialised. Further studies including retention periods or transfer to other types of tasks are needed to show the long-lasting effects and the potential use for team performance.

Regarding the team task, it may be argued that the cognitive load was quite low, as environmental complexity and degrees of freedom were reduced in our experimental setting (e.g., number of players, missing opponents, reduced decision-making). None-the-less, the task was football-specific and carried out with experienced youth football players. Therefore, we assume good external validity as a strength of this study. At the same time, because of not controlling every team dynamics factor (except team age/familiarity), internal validity may have suffered, which is a potential limitation. Other limitations pertain to the small statistical power regarding the correlational analysis and the self-constructed questions, which may have led to arguable results. Although we wanted to fulfill the premise of Cooke et al. (2013) about context-specificity of shared knowledge, we cannot prove that our instrument was reliable. None-the-less, due to its context-specific nature, we attribute face validity to our measurement of shared knowledge. However, a larger confirmatory study with appropriate measurements and a more diverse sample is needed to substantiate these findings. Further studies in the continuation of our novel approach should also focus on even more realistic football scenarios where players are engaged on an even higher cognitive level. Higher cognitive activity is required in situations that are more open and where decisions come into play, in situations that include sub-groups instead of only dyads, or in the presence of opponents (see also Santos et al., 2018). Either way, more field studies are needed to fulfill context-specificity, although internal validity may be limited. Furthermore, and for the sake of predictive validity, it would be interesting to investigate how team cognition relates to team performance in the football or team sports context in general and even in new avenues of team sports like the aspiring esports.

## Author Contributions

All authors listed have made a substantial, direct and intellectual contribution to the work, and approved it for publication.

## Conflict of Interest Statement

The authors declare that the research was conducted in the absence of any commercial or financial relationships that could be construed as a potential conflict of interest.

## Appendix

Example of a situation-specific question with illustration.

Question: As a pass giver in the *overlapping run* and *double pass* situation, when do you play the pass? (mark appropriate answer!).

As a pass giver (player in blue), I play the pass in the *overlapping run* situation, …

**Table:**

As soon as I have received the ball and my teammate is still in the same square from where he passed me the ball.	As soon as I have received the ball and my teammate is located between the squares.	As soon as my teammate is located behind my back.	As soon as my teammate has passed my back and runs towards the square we have to play in.	As soon as my teammate has passed my back and is located just in front of the square we have to play in.	As soon as my teammate stays in the square we have to play in.
